# 3-[(3-Hydroxypropyl)amino]-1-phenyl­but-2-en-1-one

**DOI:** 10.1107/S1600536808043183

**Published:** 2008-12-24

**Authors:** T. S. Arul Jeevan, K. S. Nagaraja

**Affiliations:** a Department of Chemistry, Loyola Institute of Frontier Energy, Loyola College, Chennai 600 034, India

## Abstract

The title compound, C_13_H_17_NO_2_, has an intra­molecular N—H⋯O hydrogen bond, forming a planar six-membered ring with a mean deviation of 0.015 (5) Å from the plane. This plane makes a dihedral angle of 7.19 (8)° with the adjacent phenyl ring. Through an inter­molecular O—H⋯O hydrogen bond, the mol­ecules with their 2_1_ screw and *b*-translation equivalents form a helical chain running parallel to the *b* axis.

## Related literature

For general background, see: Morozova *et al.* (2007 ▶). For a related structure, see: Shi (2005 ▶).
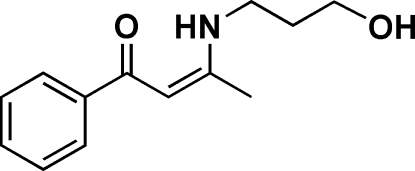


## Experimental

### 

#### Crystal data

C_13_H_17_NO_2_
*M* *_r_* = 219.28
Orthorhombic, 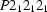

*a* = 5.9131 (3) Å
*b* = 8.0101 (4) Å
*c* = 24.9626 (13) Å
*V* = 1182.34 (10) Å^3^
*Z* = 4
Mo *K*α radiation
μ = 0.08 mm^−1^
*T* = 293 (2) K
0.30 × 0.20 × 0.20 mm

#### Data collection

Bruker Kappa APEXII CCD diffractometer
Absorption correction: multi-scan (**SADABS**; Bruker, 1999 ▶) *T*_min_ = 0.944, *T*_max_ = 0.984
11541 measured reflections
1236 independent reflections
1168 reflections with *I* > 2σ(*I*)
*R*_int_ = 0.022

#### Refinement

*R*[*F* ^2^ > 2σ(*F* ^2^)] = 0.028
*wR*(*F* ^2^) = 0.079
*S* = 1.05
1236 reflections
153 parameters
H atoms treated by a mixture of independent and constrained refinement
Δρ_max_ = 0.12 e Å^−3^
Δρ_min_ = −0.09 e Å^−3^

### 

Data collection: *APEX2* (Bruker, 2004 ▶); cell refinement: *SAINT-Plus* (Bruker, 2004 ▶); data reduction: *SAINT-Plus*; program(s) used to solve structure: *SIR92* (Altomare *et al.*, 1994 ▶); program(s) used to refine structure: *SHELXL97* (Sheldrick, 2008 ▶); molecular graphics: *ORTEP-3* (Farrugia, 1997 ▶) and *Mercury* (Macrae *et al.*, 2006 ▶); software used to prepare material for publication: *SHELXL97*.

## Supplementary Material

Crystal structure: contains datablocks global, I. DOI: 10.1107/S1600536808043183/is2371sup1.cif

Structure factors: contains datablocks I. DOI: 10.1107/S1600536808043183/is2371Isup2.hkl

Additional supplementary materials:  crystallographic information; 3D view; checkCIF report

## Figures and Tables

**Table 1:** Hydrogen-bond geometry (Å, °)

*D*—H⋯*A*	*D*—H	H⋯*A*	*D*⋯*A*	*D*—H⋯*A*
O2—H2*A*⋯O1^i^	0.82	1.99	2.805 (2)	176
N1—H1*N*⋯O1	0.85 (2)	1.94 (2)	2.642 (2)	139.1 (18)

Symmetry code: (i) 
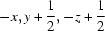
.

**Fig. 1.**
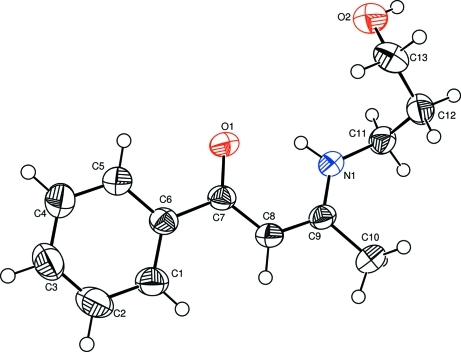
The ORTEP representation of the molecule with atoms represented as 50% probability ellipsoid.

**Fig. 2.**
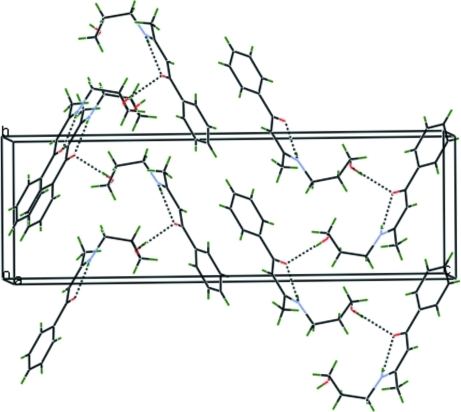
Packing of molecules in the unit cell. Intra and intermolecular interactions are shown with dotted lines.

## supplementary crystallographic information

### Comment

The Schiff base 1-phenyl-3-[(3-hydroxypropyl)amino]-1-butanone could be a good chelating ligand and may find use in the field of coordination chemistry of transition metal complexes. The compound could act as a bidentate ligand through the N and O atoms. The replacement of oxygen by nitrogen in the ligand can increase the covalency of the complexes (Morozova *et al.*, 2007).

Figure 1 gives *ORTEP* representation of the molecule with atoms represented as 50% anisotropic ellipsoids. Figure 2 gives packing of the molecules showing hydrogen bonded interactions. The molecules and their 2_1_ screw translation equivalents are bound through O2—H2A···O1 hydrogen bonds (Table 1). These H-bonded pairs are further linked with their b-translation equivalents to form an one-dimensional hydrogen bonded network parallel to *b* axis. There is an intramolecular N1—H1···O1 hydrogen bond between the imino hydrogen and the keto oxygen (Table 1).  The packing is further stabilized through van der Waals interactions. The crystal is found to cleave easily through the (001) plane. The closely related compound, C_12_H_15_O_2_N, (3-[(2-hydroxyethyl)amino]-1-phenylbut-2-en-1-one) crystallizes in monoclinic system with centrosymmetric space group *P*2_1_/*n*, forming hydrogen bonded dimers in the structure (Shi, 2005), while the title compound crystallizes in polar space group *P*2_1_2_1_2_1_ and with extended hydrogen bonding in the structure.

### Experimental

The title Schiff base ligand was synthesized by the condensation of 3-amino-1-propanol and benzoylacetone. To 0.1 molar solution of 3-amino-1-propanol (dissolved in 5 ml of ethanol) was slowly added to a 0.1 molar solution (in ethanol) of benzoylacetone. The reaction mixture was refluxed for 30 min. The solution was cooled overnight and the precipitate was washed with ethanol. The compound was crystallized in ethanol by slow evaporation (m.p. = 394 K). Anal. Calc. for C_13_H_18_O_2_N: (Found %): C 70.88 (69.96), H 8.24 (8.13), N 6.35 (6.26). IR (KBr, cm^-1^): 3170 = v(O—H); 3340, v(N—H); 1596, v[(C—N)—C=C)]; 1265, v(C—O). ^1^H NMR (400 MHz, CDCl_3_) δ values at 1.8, 2.0, 3.4, 3.6, 5.7 and 7.4 p.p.m. for CH_3_, CH_2_, NH—CH_2_, CH_2_—OH, H—C=C and aromatic protons respectively. ^13^C NMR (400 MHz, CDCl_3_) δ values at 14.83, 32.53, 40.06, 92.46, 128, 140.39 and 187 for CH_3_, CH_2_,-HN—CH_2_, –CH_2_OH, Aromatic, C—CH=C– and C=O carbon respectively. Mass Spectra: *M*+, m/*z* = 220.29, (I = 19%); *M* [*L*-(O—CH_2_—CH_2_—CH_2_—N)]+, 148.10, (22); *M* [(C_6_H_5_-C=O)]+, 104.52, (100); *M* [(O—CH_2_—CH_2_—CH_2_—N)]+, 74.63, (65).

### Refinement

All the hydrogen atoms could be located in a difference Fourier map. However, the H atoms except that of NH, were fixed at geometrically meaningful positions and refined using riding model. The riding tertiary CH_3_ hydrogen atoms were assigned 1.5 times the equivalent displacement parameters of parent atoms, while 1.2 times was assigned for CH_2_ and aromatic H atoms. The aromatic C—H distances were fixed at 0.93 Å while the secondary CH_2_ and tertiary CH_3_ were assigned 0.97 Å and 0.96 Å respectively. The isotropic displacement parameter of hydroxyl hydrogen was refined. In the absence of significant anomalous scattering effects, Friedel pairs have been merged.

### Crystal data

**Table:**

C_13_H_17_NO_2_	*F*(000) = 472
*M**_r_* = 219.28	*D*_x_ = 1.232 Mg m^−^^3^
Orthorhombic, *P*2_1_2_1_2_1_	Mo *K*α radiation, λ = 0.71073 Å
Hall symbol: P 2ac 2ab	Cell parameters from 7505 reflections
*a* = 5.9131 (3) Å	θ = 2.5–31.0°
*b* = 8.0101 (4) Å	µ = 0.08 mm^−^^1^
*c* = 24.9626 (13) Å	*T* = 293 K
*V* = 1182.34 (10) Å^3^	Needle, colourless
*Z* = 4	0.30 × 0.20 × 0.20 mm

### Data collection

**Table:**

Bruker Kappa APEXII CCD diffractometer	1236 independent reflections
Radiation source: fine-focus sealed tube	1168 reflections with *I* > 2σ(*I*)
graphite	*R*_int_ = 0.022
ω and φ scan	θ_max_ = 25.0°, θ_min_ = 2.7°
Absorption correction: multi-scan (*SADABS*; Bruker, 1999)	*h* = −7→5
*T*_min_ = 0.944, *T*_max_ = 0.984	*k* = −9→9
11541 measured reflections	*l* = −29→28

### Refinement

**Table:**

Refinement on *F*^2^	Secondary atom site location: difference Fourier map
Least-squares matrix: full	Hydrogen site location: inferred from neighbouring sites
*R*[*F*^2^ > 2σ(*F*^2^)] = 0.028	H atoms treated by a mixture of independent and constrained refinement
*wR*(*F*^2^) = 0.079	*w* = 1/[σ^2^(*F*_o_^2^) + (0.0436*P*)^2^ + 0.1578*P*] where *P* = (*F*_o_^2^ + 2*F*_c_^2^)/3
*S* = 1.05	(Δ/σ)_max_ < 0.001
1236 reflections	Δρ_max_ = 0.12 e Å^−^^3^
153 parameters	Δρ_min_ = −0.09 e Å^−^^3^
0 restraints	Extinction correction: *SHELXL*, Fc^*^=kFc[1+0.001xFc^2^λ^3^/sin(2θ)]^-1/4^
Primary atom site location: structure-invariant direct methods	Extinction coefficient: 0.025 (3)

### Special details

**Table:**

Geometry. All e.s.d.'s (except the e.s.d. in the dihedral angle between two l.s. planes) are estimated using the full covariance matrix. The cell e.s.d.'s are taken into account individually in the estimation of e.s.d.'s in distances, angles and torsion angles; correlations between e.s.d.'s in cell parameters are only used when they are defined by crystal symmetry. An approximate (isotropic) treatment of cell e.s.d.'s is used for estimating e.s.d.'s involving l.s. planes.
Refinement. Refinement of *F*^2^ against ALL reflections. The weighted *R*-factor *wR* and goodness of fit *S* are based on *F*^2^, conventional *R*-factors *R* are based on *F*, with *F* set to zero for negative *F*^2^. The threshold expression of *F*^2^ > σ(*F*^2^) is used only for calculating *R*-factors(gt) *etc*. and is not relevant to the choice of reflections for refinement. *R*-factors based on *F*^2^ are statistically about twice as large as those based on *F*, and *R*- factors based on ALL data will be even larger.

### Fractional atomic coordinates and isotropic or equivalent isotropic displacement parameters (Å^2^)

**Table:**

	*x*	*y*	*z*	*U*_iso_*/*U*_eq_	
C1	0.5484 (4)	−0.3200 (2)	0.04625 (8)	0.0547 (5)	
H1	0.6664	−0.2439	0.0430	0.066*	
C2	0.5575 (4)	−0.4692 (3)	0.01828 (8)	0.0652 (6)	
H2	0.6805	−0.4922	−0.0037	0.078*	
C3	0.3864 (4)	−0.5822 (3)	0.02298 (8)	0.0647 (6)	
H3	0.3928	−0.6825	0.0043	0.078*	
C4	0.2058 (4)	−0.5480 (3)	0.05513 (8)	0.0664 (6)	
H4	0.0900	−0.6258	0.0586	0.080*	
C5	0.1939 (4)	−0.3984 (3)	0.08253 (7)	0.0554 (5)	
H5	0.0680	−0.3753	0.1036	0.066*	
C6	0.3664 (3)	−0.2826 (2)	0.07901 (6)	0.0410 (4)	
C7	0.3439 (3)	−0.1211 (2)	0.10934 (6)	0.0400 (4)	
C8	0.5240 (3)	−0.0080 (2)	0.11068 (6)	0.0428 (4)	
H8	0.6541	−0.0342	0.0915	0.048 (5)*	
C9	0.5197 (3)	0.1415 (2)	0.13909 (6)	0.0409 (4)	
C10	0.7269 (3)	0.2485 (3)	0.14091 (9)	0.0608 (5)	
H9A	0.6929	0.3566	0.1264	0.091*	
H9B	0.8447	0.1973	0.1201	0.091*	
H9C	0.7762	0.2601	0.1774	0.091*	
C11	0.3128 (3)	0.3395 (2)	0.19816 (7)	0.0489 (5)	
H10A	0.1609	0.3829	0.1936	0.059*	
H10B	0.4180	0.4238	0.1857	0.059*	
C12	0.3543 (3)	0.3077 (3)	0.25711 (7)	0.0560 (5)	
H11A	0.5089	0.2699	0.2618	0.067*	
H11B	0.3377	0.4120	0.2765	0.067*	
C13	0.1971 (4)	0.1805 (3)	0.28110 (7)	0.0575 (5)	
H12A	0.2223	0.0735	0.2639	0.069*	
H12B	0.2318	0.1679	0.3189	0.069*	
N1	0.3385 (3)	0.19014 (19)	0.16559 (6)	0.0438 (4)	
O1	0.1589 (2)	−0.09436 (16)	0.13305 (5)	0.0543 (4)	
O2	−0.0328 (2)	0.2249 (2)	0.27548 (6)	0.0679 (4)	
H2A	−0.0723	0.2808	0.3014	0.099 (10)*	
H1N	0.227 (3)	0.124 (3)	0.1625 (8)	0.049 (5)*	

### Atomic displacement parameters (Å^2^)

**Table:**

	*U*^11^	*U*^22^	*U*^33^	*U*^12^	*U*^13^	*U*^23^
C1	0.0546 (11)	0.0488 (10)	0.0607 (10)	−0.0008 (10)	0.0154 (9)	0.0000 (9)
C2	0.0728 (14)	0.0592 (12)	0.0638 (12)	0.0068 (12)	0.0237 (12)	−0.0057 (10)
C3	0.0883 (16)	0.0500 (11)	0.0559 (10)	−0.0010 (12)	0.0091 (12)	−0.0107 (9)
C4	0.0767 (16)	0.0610 (12)	0.0615 (11)	−0.0212 (12)	0.0108 (12)	−0.0123 (10)
C5	0.0540 (11)	0.0626 (11)	0.0495 (9)	−0.0137 (11)	0.0107 (9)	−0.0108 (9)
C6	0.0435 (9)	0.0453 (9)	0.0341 (7)	−0.0013 (8)	0.0018 (7)	0.0036 (7)
C7	0.0389 (9)	0.0474 (9)	0.0338 (7)	−0.0013 (8)	0.0041 (7)	0.0029 (7)
C8	0.0386 (9)	0.0511 (10)	0.0388 (8)	−0.0040 (8)	0.0076 (8)	−0.0018 (7)
C9	0.0359 (9)	0.0509 (9)	0.0359 (7)	−0.0064 (8)	0.0011 (7)	0.0042 (7)
C10	0.0451 (11)	0.0699 (13)	0.0674 (11)	−0.0171 (10)	0.0066 (9)	−0.0082 (11)
C11	0.0467 (10)	0.0418 (9)	0.0583 (9)	−0.0049 (9)	0.0044 (9)	−0.0059 (8)
C12	0.0481 (10)	0.0662 (12)	0.0538 (9)	0.0008 (11)	−0.0027 (9)	−0.0156 (9)
C13	0.0636 (13)	0.0598 (12)	0.0492 (9)	0.0125 (12)	0.0086 (9)	−0.0018 (9)
N1	0.0384 (8)	0.0449 (8)	0.0481 (7)	−0.0081 (8)	0.0042 (7)	−0.0052 (7)
O1	0.0418 (7)	0.0553 (7)	0.0659 (7)	−0.0085 (7)	0.0168 (6)	−0.0116 (6)
O2	0.0542 (9)	0.0851 (11)	0.0643 (8)	−0.0029 (9)	0.0093 (7)	−0.0059 (9)

### Geometric parameters (Å, °)

**Table:**

C1—C6	1.385 (3)	C9—C10	1.496 (2)
C1—C2	1.385 (3)	C10—H9A	0.9600
C1—H1	0.9300	C10—H9B	0.9600
C2—C3	1.363 (3)	C10—H9C	0.9600
C2—H2	0.9300	C11—N1	1.454 (2)
C3—C4	1.364 (3)	C11—C12	1.513 (2)
C3—H3	0.9300	C11—H10A	0.9700
C4—C5	1.382 (3)	C11—H10B	0.9700
C4—H4	0.9300	C12—C13	1.504 (3)
C5—C6	1.381 (3)	C12—H11A	0.9700
C5—H5	0.9300	C12—H11B	0.9700
C6—C7	1.505 (2)	C13—O2	1.412 (3)
C7—O1	1.262 (2)	C13—H12A	0.9700
C7—C8	1.399 (2)	C13—H12B	0.9700
C8—C9	1.392 (2)	N1—H1N	0.85 (2)
C8—H8	0.9300	O2—H2A	0.8200
C9—N1	1.318 (2)		
			
C6—C1—C2	120.95 (19)	C9—C10—H9B	109.5
C6—C1—H1	119.5	H9A—C10—H9B	109.5
C2—C1—H1	119.5	C9—C10—H9C	109.5
C3—C2—C1	120.07 (19)	H9A—C10—H9C	109.5
C3—C2—H2	120.0	H9B—C10—H9C	109.5
C1—C2—H2	120.0	N1—C11—C12	112.86 (16)
C2—C3—C4	119.90 (19)	N1—C11—H10A	109.0
C2—C3—H3	120.0	C12—C11—H10A	109.0
C4—C3—H3	120.0	N1—C11—H10B	109.0
C3—C4—C5	120.4 (2)	C12—C11—H10B	109.0
C3—C4—H4	119.8	H10A—C11—H10B	107.8
C5—C4—H4	119.8	C13—C12—C11	113.63 (16)
C6—C5—C4	120.85 (18)	C13—C12—H11A	108.8
C6—C5—H5	119.6	C11—C12—H11A	108.8
C4—C5—H5	119.6	C13—C12—H11B	108.8
C5—C6—C1	117.82 (16)	C11—C12—H11B	108.8
C5—C6—C7	118.66 (15)	H11A—C12—H11B	107.7
C1—C6—C7	123.48 (16)	O2—C13—C12	112.62 (18)
O1—C7—C8	122.59 (15)	O2—C13—H12A	109.1
O1—C7—C6	117.30 (15)	C12—C13—H12A	109.1
C8—C7—C6	120.12 (15)	O2—C13—H12B	109.1
C9—C8—C7	123.76 (15)	C12—C13—H12B	109.1
C9—C8—H8	118.1	H12A—C13—H12B	107.8
C7—C8—H8	118.1	C9—N1—C11	127.51 (16)
N1—C9—C8	121.66 (16)	C9—N1—H1N	113.8 (13)
N1—C9—C10	118.77 (15)	C11—N1—H1N	118.7 (13)
C8—C9—C10	119.55 (15)	C13—O2—H2A	109.5
C9—C10—H9A	109.5		
			
C6—C1—C2—C3	−0.4 (3)	C1—C6—C7—C8	−7.9 (2)
C1—C2—C3—C4	0.2 (4)	O1—C7—C8—C9	1.8 (3)
C2—C3—C4—C5	0.8 (4)	C6—C7—C8—C9	−177.97 (15)
C3—C4—C5—C6	−1.6 (3)	C7—C8—C9—N1	−2.6 (2)
C4—C5—C6—C1	1.4 (3)	C7—C8—C9—C10	175.92 (16)
C4—C5—C6—C7	179.54 (18)	N1—C11—C12—C13	−59.7 (2)
C2—C1—C6—C5	−0.4 (3)	C11—C12—C13—O2	−57.9 (2)
C2—C1—C6—C7	−178.45 (18)	C8—C9—N1—C11	177.83 (15)
C5—C6—C7—O1	−5.7 (2)	C10—C9—N1—C11	−0.7 (3)
C1—C6—C7—O1	172.33 (17)	C12—C11—N1—C9	−95.5 (2)
C5—C6—C7—C8	174.08 (16)		

### Hydrogen-bond geometry (Å, °)

**Table:**

*D*—H···*A*	*D*—H	H···*A*	*D*···*A*	*D*—H···*A*
O2—H2A···O1^i^	0.82	1.99	2.805 (2)	176
N1—H1N···O1	0.85 (2)	1.94 (2)	2.642 (2)	139.1 (18)

Symmetry codes: (i) −*x*, *y*+1/2, −*z*+1/2.

## References

[bb1] Altomare, A., Cascarano, G., Giacovazzo, C., Guagliardi, A., Burla, M. C., Polidori, G. & Camalli, M. (1994). *J. Appl. Cryst.* **27**, 435.

[bb2] Bruker (1999). *SADABS* Bruker AXS Inc., Madison, Wisconsin, USA.

[bb3] Bruker (2004). *APEX2* and *SAINT-Plus* Bruker AXS Inc., Madison, Wisconsin, USA.

[bb4] Farrugia, L. J. (1997). *J. Appl. Cryst.* **30**, 565.

[bb5] Macrae, C. F., Edgington, P. R., McCabe, P., Pidcock, E., Shields, G. P., Taylor, R., Towler, M. & van de Streek, J. (2006). *J. Appl. Cryst.* **39**, 453–457.

[bb6] Morozova, N. B., Stabnikov, P. A. & Igumenov, I. K. (2007). *J. Struct. Chem.* **48**, 889–898.

[bb7] Sheldrick, G. M. (2008). *Acta Cryst.* A**64**, 112–122. 10.1107/S0108767307043930 18156677

[bb8] Shi, Y.-C. (2005). *Acta Cryst.* E**61**, o2005–o2007.

